# Generalized div-curl based regularization for physically constrained deformable image registration

**DOI:** 10.1038/s41598-024-65896-3

**Published:** 2024-07-01

**Authors:** Paris Tzitzimpasis, Mario Ries, Bas W. Raaymakers, Cornel Zachiu

**Affiliations:** 1 Department of Radiotherapy, UMC Utrecht, 3584 CX Utrecht, The Netherlands; 2 Imaging Division, UMC Utrecht, 3584 CX Utrecht, The Netherlands

**Keywords:** Computational models, Image processing, Computational science

## Abstract

Variational image registration methods commonly employ a similarity metric and a regularization term that renders the minimization problem well-posed. However, many frequently used regularizations such as smoothness or curvature do not necessarily reflect the underlying physics that apply to anatomical deformations. This, in turn, can make the accurate estimation of complex deformations particularly challenging. Here, we present a new highly flexible regularization inspired from the physics of fluid dynamics which allows applying independent penalties on the divergence and curl of the deformations and/or their nth order derivative. The complexity of the proposed generalized div-curl regularization renders the problem particularly challenging using conventional optimization techniques. To this end, we develop a transformation model and an optimization scheme that uses the divergence and curl components of the deformation as control parameters for the registration. We demonstrate that the original unconstrained minimization problem reduces to a constrained problem for which we propose the use of the augmented Lagrangian method. Doing this, the equations of motion greatly simplify and become managable. Our experiments indicate that the proposed framework can be applied on a variety of different registration problems and produce highly accurate deformations with the desired physical properties.

## Introduction

Image registration is a challenging task with numerous applications ranging from remote sensing to astronomy and art. In particular, deformable image registration is a topic of major interest in medical image analysis^1^. Given two images obtained at different times, with different devices or even from different scenes, the objective is to establish a spatial transformation between them, whereby the term “images” refers generally to 2D as well as 3D information. In general, this can be formulated as the minimization of a distance metric which quantifies the degree of similarity (or dissimilarity) between the given images. Various such metrics have been studied in the literature such as the sum of squared differences (SSD), mutual information^2,3^, normalized gradient fields^4^ and others^1^.

Due to the inherent ill-posedness of the problem in the sense of Hadamard^5^, the optimization of a data similarity metric alone leads to unstable and non-smooth solutions. To aleviate this, variational methods generally introduce a regularization term in the minimization functional to ensure well-posedness and restrict the solution space to transformations that conform with certain desirable properties. The diffusion regularization^6^ serves as a straightforward approach to avoid oscillatory and non-smooth solutions. The curvature regularization^7,8^ provides similar benefits without penalizing rigid motion while combinations of the two^9^ can offer benefits in terms of registration accuracy and deformation field plausibility. The elastic potential^10^ employs a model stemming from the theory of linear elasticity but is a valid approximation only when the deformations are sufficiently small. To address this, the hyperelastic potential has been proposed^11^, which takes into account the non-linear properties of the deformations and also penalizes non-diffeomorphic deformations. The fluid regularization^12^ has been proposed as a method to address large scale initial deformations while maintaining the continuity of anatomical structures by using a non-linear transformation model and a regularization inspired from fluid dynamics. The rigidity penalty^13^ is a local penalty term aiming to constrain the solution space and enforce some physical prior expectation such as tumor volume preservation. The total variation (TV) regularization^14^ has been a notably efficient method for allowing vector field discontinuities and modeling sliding motion. In particular, the isotropic total variation^15^ has been demonstrated to be well-suited for lung image registration, achieving optimal performance on a number of public datasets. Recently, the arbitrary order total variation regularization^16^ has provided a generalized framework involving higher-order derivatives. Finally, the locally adaptive total p-variation (LaTpV)^17^ has been introduced to selectively preserve discontinuities at organ boundaries while penalizing them inside organs using organ segmentations.

To date, the large collection of different regularization penalties paired together with a multitude of available distance metrics have allowed to address a large variety of medical image registration problems very successfully. Nevertheless, one drawback of such a broad canon of combinations is that the solutions are generally insular with respect to their respective applicability and that each combination introduces their own respective numerical challenges. For example, the non-differentiability of TV renders the optimization of TV-based functionals challenging, which can introduce numerical instabilities in the optimization process^16^. Additionally, TV based regularization methods rely on heuristic principles and despite delivering excellent results on sliding surfaces such as organ boundaries, the estimated deformations in the interior of anatomical regions are not derived from explicit physical models, which complicates the interpretability of the results in regions devoid of image contrast. On the other hand, regularization penalties that are based on physical models such as the fluid regularization admit a physical interpretation but suffer from numerical instabilities that can lead to grid foldings. To ameliorate this, numerical stability methods such as regridding or smoothing are often employed^12^. Other physically motivated regularizations such as the hyperelastic model have been evaluated on clinically relevant scenarios^18^ with success, but so far not found widespread clinical adoption. Ideally, a regularization penalty should both be adaptable to a large variety of different tissue types and application scenarios and physically motivated. Nevertheless, despite considerable effort in the recent years, such a regularization penalty providing the “best of both worlds” has remained elusive. Here, we like to present a promising candidate for such a regularization and evaluate it’s properties and advantages on a broad set of medical image registration tasks.

A significant amount of research has been devoted to the construction of realistic transformation models for image registration that exclude some highly unphysical deformations and can serve as implicit regularization methods. Such methods include B-splines^19^, volume preserving transformations^20^ or viscous elastic models and diffeomorphic vector fields^21^. While these methods have demonstrated substantial merit and potential for specific anatomies/deformations, their respective generic applicability is frequently limited by their specific assumptions about the underlying physics. For example, volume preserving methods are only well-suited for incompressible anatomies while diffeomorphic fields are not a valid assumption in the presence of absent correspondences and disappearing structures such as inter-subject registration or registration of pre-operative to post-operative scans^22^. An alternative representation of the deformation fields can be given by means of the Helmholtz decomposition which expresses a vector field in terms of its divergence and curl components. Using this approach, the registration parameters admit a clear physical interpretation. This is to be contrasted with the traditionally used Cartesian vector field components which are practical but coordinate dependent. The Helmholtz parametrization has been used^23–26^ to minimize a similarity metric with no explicit regularization, taking advantage of the smoothness inherited from the transformation model. It has been reported^24^ that using such an implicit representation results in better stability than B-splines while avoiding grid foldings. A decomposition in terms of potential functions for the divergence and curl has also been proposed^27^ in conjunction with more complex regularizers. In the present work, we use the rationale of this prior art to optimize a functional consisting of a similarity metric and a regularization which controls certain physical properties of the estimated motion fields. The contribution of our work can be summarized in the following points:

- We introduce a novel generalized regularizer that is expressed in terms of the divergence and curl, thus admitting a clear physical interpretation. Our regularizer is highly versatile, allowing for adaptation to problems with different underlying physical principles.
- The optimization is expressed in terms of the divergence and curl using the Helmholtz decomposition. Although this decomposition has been used before, to our knowledge, this is the first work that introduces a constraint to ensure that the curl vector field has vanishing divergence. This constraint is crucial to allow for a physical interpretation of the divergence and curl (in the 3D case) as we explain below.
- We provide a numerical scheme that allows for an efficient solution irrespective of the regularizer complexity. This is highly desirable since complex physical models are often numerically unstable and computationally heavy, especially when higher-order terms need to be included.

## Methods

We state our image registration problem, which incorporates a regularization term penalizing an arbitrary order of the divergence and curl operators of the estimated deformation field. Given the fixed and moving images ${\mathscr {F}},{\mathscr {M}}: \Omega \rightarrow {\mathbb {R}}$ defined on some domain $\Omega \subset {\mathbb {R}}^3$, we seek a deformation field $\phi : \Omega \rightarrow \Omega$, such that $M \circ \phi$ is aligned with *F*. The deformation field is usually expressed as $\phi (\vec {x}) = \vec {x} + \vec {u} (\vec {x})$ where $\vec {u} (\vec {x})$ is the displacement or motion field. We can then formulate image registration as an optimization problem where the optimal displacement field $\vec {u} ^*$ is given by:

1 $$\begin{aligned} \arg \min _{ u^i } \; \bigg \{ \int _{\Omega } {\mathscr {D}}({\mathscr {F}},{\mathscr {M}},\vec {u})+ \alpha \left\| \nabla ^{\sigma } \text {div} \; \vec {u} \right\| ^2 + \beta \left\| \nabla ^{\kappa } \text {curl} \; \vec {u} \right\| ^2 \; d \vec {x} \bigg \}, \end{aligned}$$

where $\Omega$ denotes the image domain, $u^i(\vec {x}): \Omega \rightarrow {\mathbb {R}}^n$ are the vector field components, $\vert \vert \cdot \vert \vert$ is the standard Euclidean norm in ${\mathbb {R}}^3$, ${\mathscr {D}}$ denotes the image similarity metric, $\alpha$ and $\beta$ are regularization weights and $\kappa ,\sigma$ are non-negative integers. The functional 1 generalizes previously studied regularizers. In particular, we distinguish the following special cases:

- For $\kappa = \sigma = 0$ we recover the first order div-curl regularizer that penalizes the amplitude of the divergence and curl of the estimated vector fields^28,29^. For $\alpha = \beta$ this model is equivalent to the diffusion (smoothness) regularization, at the level of the Euler-Lagrange equations^30^. This equivalence also demonstrates the highly restrictive nature of the diffusion regularization which equally penalizes two quantities, the divergence and curl, that have no a priori reason to be treated evenly. Another disadvantage of this regularizer is a tendency to underestimate the flow gradients^31^.
- For $\kappa = \sigma =1$ Eq. (1) reduces to the second order div-curl model^25,29,30,32,33^. This model enforces smoothness on the divergence and curl components without penalizing the components themselves. Although this description is not derived as an approximation to fundamental physics laws, it forces the divergence and curl quantities to form coherent aggregates, thus abiding by the physical properties of fluid flows^34^. The numerical implementation is considerably more demanding than its first order counterpart due to the higher order derivatives involved. One method to overcome this is the introduction of auxiliary variables that approximate $\text {div} \,\vec {u}$ and $\text {curl} \,\vec {u}$ by means of additional constraints^25,29^. However, as we will show, in our generalized framework the computational complexity is not affected by the order that one chooses to work with. This constitues a substantial advantage of our approach.

### Optimization scheme

We now proceed to the description of the optimization method employed to solve Eq. (1). The starting point is the adoption of canonical variables. Instead of the conventional Cartesian components $u^i(\vec {x}) ,\; i=1,2,3$ of the vector field $\vec {\text {v}}$, we select the divergence $f^1(\vec {x}) = \text {div} \,\vec {\text {u}}$ and the curl components $f^i (\vec {x}) = (\text {curl} \; \vec {u})^{i-1}$ with $i=2,3,4$. The key observation behind this strategy, is the ability to go back and forth between $u^i(\vec {x})$ and $f^i(\vec {x})$ which has been utilized in previous works^23,24,26^. The forward mapping $f^i = G(u^i)$ is straightforward and follows from the definition of the $f^i$:

$$\begin{aligned}{}&f^1(\vec {x}) = \text {div} \,\vec {\text {u} } = \frac{\partial u^1(\vec {x})}{\partial x} + \frac{\partial u^2(\vec {x})}{\partial y} +\frac{\partial u^3(\vec {x})}{\partial z}, \\&f^2(\vec {x}) = (\text {curl} \,\vec {\text {u} })^1 = \frac{\partial u^3(\vec {x})}{\partial y} - \frac{\partial u^2(\vec {x})}{\partial z}, \\&f^3(\vec {x}) = (\text {curl} \,\vec {\text {u} })^2 = \frac{\partial u^1(\vec {x})}{\partial z} - \frac{\partial u^3(\vec {x})}{\partial x}, \\&f^4(\vec {x}) = (\text {curl} \,\vec {\text {u} })^3 = \frac{\partial u^2(\vec {x})}{\partial x} - \frac{\partial u^1(\vec {x})}{\partial y}. \end{aligned}$$

The existence of an inverse mapping $u^i = F(f^i)$ is a consequence of the Helmholtz theorem which states that given a solenoidal vector field $\vec {C}$ (so that $\nabla \cdot \vec {C} = 0$) and a scalar field $\Phi$ that are sufficiently smooth and vanish faster than $1/r^2$ at infinity, there exists a vector field $\vec {v}$ such that

2 $$\begin{aligned} \nabla \times \vec {v} = \vec {C} \;\;\; , \;\;\; \nabla \cdot \vec {v} = \Phi , \end{aligned}$$

which is unique provided that $|| \vec {v} || \rightarrow 0$ as $r \rightarrow \infty$. The inverse mapping $u^i = F(f^i)$ is implicit and given by solving the following set of Poisson equations

3 $$\begin{aligned} \Delta u^1 = \frac{\partial f^1}{\partial x} + \frac{\partial f^3}{\partial z} - \frac{\partial f^4}{\partial y} =: F^1 \;\;\;\; , \;\;\;\;\;\; \Delta u^2 = \frac{\partial f^1}{\partial y} + \frac{\partial f^4}{\partial x} - \frac{\partial f^2}{\partial z} =: F^2 \;\;\;\; , \;\;\;\;\;\; \Delta u^3 = \frac{\partial f^1}{\partial z} + \frac{\partial f^2}{\partial y} - \frac{\partial f^3}{\partial x} =: F^3 , \end{aligned}$$

where $\Delta$ is the Laplace operator. There exist various div-curl solvers for solving Eq. (3) using direct discretization, successive over relaxation, inverse filter and FFT transformations. As has been previously pointed out^24^, the later provides both accuracy and computational efficiency of ${\mathcal {O}}(n \log n$) complexity. The FFT-solver operates on the assumption that the null boundary condition is fulfilled. For this reason we adopted the FFT-solver for this work. An important caveat in this discussion is that in order to ensure that the maps *F* and *G* are inverses of each other, the curl components $f^i ,\; i=2,3,4$ must satisfy the following differential constraint:

4 $$\begin{aligned} {\mathscr {C}}(\vec {x}) := \frac{\partial f^2 ( \vec {x})}{ \partial x} + \frac{\partial f^3 ( \vec {x})}{ \partial y} + \frac{\partial f^4 ( \vec {x})}{ \partial z} =0 , \end{aligned}$$

which is a consequence of the general identity $\nabla \cdot \nabla \times \vec {h} =0$ for any differentiable vector field $h \in {\mathfrak {X}}^2({\mathbb {R}}^3)$. Viewed differently, this is also the requirement that the vector field $\vec {C}$ in Eq. (2) is solenoidal. The existence of a constraint equation should also be expected due to the mismatch of the number of free parameters in the two pictures. Using the traditional parametrization to describe motion, there are three degrees of freedom per voxel to be estimated, corresponding to the three Cartesian components of the deformation field. In contrast, in the proposed framework this number increases to four parameters per voxel, namely the three curl components together with the divergence. Since both are equivalent ways of parametrizing motion, the information required should be the same. This apparent paradox is resolved by the existence of the constraint Eq. (4) which reduces the effective curl-related degrees of freedom to two. In view of the established equivalence, we recast the minimization problem 1 as

5 $$\begin{aligned}{}&(f^i)^* = \arg \min _{f^i} \; \bigg \{ {\mathscr {J}}(f^i) := \int {\mathscr {D}}({\mathscr {F}},{\mathscr {M}},f^i) + \alpha \left\| \nabla ^{\sigma } f^1 ( \vec {x}) \; \right\| ^2 + \beta \sum _{j=2} ^4 \left\| \nabla ^{\kappa } f^j ( \vec {x}) \right\| ^2 \; d \vec {x} \bigg \} , \nonumber \\&\text {s.t. } \frac{\partial f^2 ( \vec {x})}{ \partial x} + \frac{\partial f^3 ( \vec {x})}{ \partial y} + \frac{\partial f^4 ( \vec {x})}{ \partial z} =0 \;\; \text {(solenoidal constraint).} \end{aligned}$$

We have in this way considerably simplified the form of our regularization at the cost of introducing an equality constraint. Although past studies have explored the use of the Helmholtz decomposition for image registration, to our knowledge this is the first work where the solenoidal constraint is introduced. In the absence of this constraint, the minimization problem can still be well defined but $f^i$ will not admit the physical interpretation of the divergence and curl components and therefore the optimization problems 5 and 1 would not be equivalent. It is also interesting to note that in 2D the curl of a vector field only has one component and the equivalence between the two pictures holds without the inclusion of any constraint. In this work, our focus is 3D registration and we are consequently led to employ constrained optimization techniques. Hence, we proceed by using the Augmented Lagrangian Method (ALM) to rewrite 5 as an unconstrained minimization problem. This method is similar to the penalty method where the constraints are directly incorporated into the minimization functional using a penalty weight. In contrast with the penalty method though, the ALM introduces additional dynamical variables to the objective function (Lagrange multipliers) that are used to enforce the constraints. This way, the ALM offers typically faster convergence speed and reduced sensitivity to the initial choice of parameters (penalty weight). For those reasons, the method has been gaining some ground in the field of image processing and has been successfully applied in image registration methods^35^. For the minimization problem 5, the augmented Lagrangian is given by

6 $$\begin{aligned} {\mathscr {L}} = {\mathscr {J}} (f^i) + \frac{\theta }{2} \int \bigg ( \frac{\partial f^2}{ \partial x} + \frac{\partial f^3}{ \partial y} + \frac{\partial f^4}{ \partial z} \bigg )^2 d \vec {x} + \int \lambda ( \vec {x} ) \bigg ( \frac{\partial f^2}{ \partial x} + \frac{\partial f^3}{ \partial y} + \frac{\partial f^4}{ \partial z} \bigg ) d \vec {x}, \end{aligned}$$

where ${\mathscr {J}} (f^i)$ is defined in 5, $\theta$ is the penalty weight and $\lambda$ is the Lagrange multiplier. The Euler-Lagrange equations follow

7 $$\begin{aligned}{}&\frac{\delta {\mathscr {L}}}{\delta f^1} =\frac{\delta {\mathscr {D}}}{\delta f^1} + \alpha {\mathscr{A}} ^{\sigma } f^1 = 0, \nonumber \\&\frac{\delta {\mathscr {L}}}{\delta f^2} =\frac{\delta {\mathscr {D}}}{\delta f^2} + \beta {\mathscr {A}} ^{\kappa } f^2 - \theta \frac{\partial }{\partial x} \bigg ( \frac{\partial f^2}{ \partial x} + \frac{\partial f^3}{ \partial y} + \frac{\partial f^4}{ \partial z} \bigg ) - \frac{\partial \lambda }{\partial x} = 0 , \nonumber \\&\frac{\delta {\mathscr {L}}}{\delta f^3} =\frac{\delta {\mathscr {D}}}{\delta f^3} + \beta {\mathscr {A}} ^{\kappa } f^3 - \theta \frac{\partial }{\partial y} \bigg ( \frac{\partial f^3}{ \partial x} + \frac{\partial f^3}{ \partial y} + \frac{\partial f^4}{ \partial z} \bigg ) - \frac{\partial \lambda }{\partial y} = 0 , \nonumber \\&\frac{\delta {\mathscr {L}}}{\delta f^4} =\frac{\delta {\mathscr {D}}}{\delta f^4} + \beta {\mathscr{A}} ^{\kappa } f^4 - \theta \frac{\partial }{\partial z} \bigg ( \frac{\partial f^2}{ \partial x} + \frac{\partial f^3}{ \partial y} + \frac{\partial f^4}{ \partial z} \bigg ) - \frac{\partial \lambda }{\partial z} = 0 , \end{aligned}$$

where ${\mathcal {A}}^{\rho }$ denotes the differential operator that corresponds to the variation of the $\left\| \nabla ^{\rho } f^i \; \right\| ^2$ term. An efficient method for dealing with such equations has been previously presented^9,36^ where a semi-implicit iterative scheme is used. In this scheme, the linear terms ${\mathcal {A}}^{\rho }f^i$ are treated implicitly and the non-linear similarity metric gradient is treated explicitly. The equations of motion 7 are embedded in a time-marching scheme and a steady-state solution is sought. Using *k* to denote the iteration number and *t* the time step we have

8 $$\begin{aligned}{}&f^1_{k+1}(\vec {x}) = \text {IDCT} \bigg \{ (1+\alpha t \Lambda ^{\sigma } _{j_1,j_2,j_3})^{-1} \text {DCT} \bigg \{ f^1 _k(\vec {x}) - t\bigg (\frac{\delta {\mathscr {D}}}{\delta f^1} \bigg )_k(\vec {x}) \bigg \} \bigg \}, \nonumber \\&f^2_{k+1}(\vec {x}) = \text {IDCT} \bigg \{ (1+\beta t \Lambda ^{\kappa } _{j_1,j_2,j_3})^{-1} \text {DCT} \bigg \{ f^2 _k (\vec {x}) - t\bigg (\frac{\delta {\mathscr {D}}}{\delta f^2} \bigg )_k (\vec {x})- t \theta \frac{\partial }{\partial x} \bigg ( \frac{\partial f^2_k(\vec {x})}{ \partial x} + \frac{\partial f^3_k(\vec {x})}{ \partial y} + \frac{\partial f^4_k(\vec {x})}{ \partial z} \bigg ) - t\frac{\partial \lambda _k (\vec {x})}{\partial x} \bigg \} \bigg \}, \nonumber \\&f^3_{k+1}(\vec {x}) = \text {IDCT} \bigg \{ (1+\beta t \Lambda ^{\kappa } _{j_1,j_2,j_3})^{-1} \text {DCT} \bigg \{ f^3 _k(\vec {x}) - t\bigg (\frac{\delta {\mathscr {D}}}{\delta f^3} \bigg )_k (\vec {x}) - t \theta \frac{\partial }{\partial y} \bigg ( \frac{\partial f^2_k (\vec {x})}{ \partial x} + \frac{\partial f^3_k(\vec {x})}{ \partial y} + \frac{\partial f^4_k(\vec {x})}{ \partial z} \bigg ) - t\frac{\partial \lambda _k (\vec {x})}{\partial y} \bigg \} \bigg \}, \nonumber \\&f^4_{k+1}(\vec {x}) = \text {IDCT} \bigg \{ (1+\beta t \Lambda ^{\kappa } _{j_1,j_2,j_3})^{-1} \text {DCT} \bigg \{ f^4 _k (\vec {x}) - t\bigg (\frac{\delta {\mathscr {D}}}{\delta f^4} \bigg )_k(\vec {x}) - t \theta \frac{\partial }{\partial z} \bigg ( \frac{\partial f^2_k(\vec {x})}{ \partial x} + \frac{\partial f^3_k(\vec {x})}{ \partial y} + \frac{\partial f^4_k(\vec {x})}{ \partial z} \bigg ) - t\frac{\partial \lambda _k (\vec {x})}{\partial z} \bigg \} \bigg \}, \end{aligned}$$

where DCT and IDCT are the discrete cosine transform and its inverse and

$$\begin{aligned} \Lambda _{j_1,j_2,j_3} ^{\rho } = \bigg ( 6 - \sum _{m=1} ^3 2 \cos \frac{(j_m-1)\pi }{n_m} \bigg )^{\rho }, \end{aligned}$$

are the eigenvalues of the operator ${\mathcal {A}}^{\rho }$ and $j_m = 1,...,n_m$ with $n_m$ denoting the image dimension in the $m{\text {th}}$ coordinate. The only remaining ambiguity in the above equations is the computation of the similarity metric gradients $\delta {\mathscr {D}}/\delta f^i$. Those terms are computed using the chain rule^24^. The process is decomposed into two steps, where first one computes

$$\begin{aligned} \frac{\delta {\mathscr {D}}}{\delta F^i} = \text {FFT-solver} \bigg ( \frac{ \delta {\mathscr {D}}}{\delta u^i} \bigg ) \;\;\;\;\; i=1,2,3 , \end{aligned}$$

where $F^i$ are the inhomogeneous parts of the Poisson equations defined in Eq. 3. At a second step, the quantities of interest are computed as follows

9 $$\begin{aligned}{}&\frac{ \delta {\mathscr {D}}}{\delta f^1} = \frac{\partial }{\partial x} \bigg ( \frac{\delta {\mathscr {D}}}{\delta F^1} \bigg ) + \frac{\partial }{\partial y} \bigg ( \frac{\delta {\mathscr {D}}}{\delta F^2} \bigg ) +\frac{\partial }{\partial z} \bigg ( \frac{\delta {\mathscr {D}}}{\delta F^3} \bigg ), \nonumber \\&\frac{ \delta {\mathscr {D}}}{\delta f^2} = -\frac{\partial }{\partial z} \bigg ( \frac{\delta {\mathscr {D}}}{\delta F^2} \bigg ) + \frac{\partial }{\partial y} \bigg ( \frac{\delta {\mathscr {D}}}{\delta F^3} \bigg ), \nonumber \\&\frac{ \delta {\mathscr {D}}}{\delta f^3} = \frac{\partial }{\partial z} \bigg ( \frac{\delta {\mathscr {D}}}{\delta F^1} \bigg ) -\frac{\partial }{\partial x} \bigg ( \frac{\delta {\mathscr {D}}}{\delta F^3} \bigg ), \nonumber \\&\frac{ \delta {\mathscr {D}}}{\delta f^4} = -\frac{\partial }{\partial y} \bigg ( \frac{\delta {\mathscr {D}}}{\delta F^1} \bigg ) +\frac{\partial }{\partial x} \bigg ( \frac{\delta {\mathscr {D}}}{\delta F^2} \bigg ). \end{aligned}$$

### Implementation details

In this work, we used the Local Correlation Coefficient (LCC) metric^37^ which generalizes the cross-correlation similarity metric by assuming a linear relationship between image intensities only locally. The image similarity gradient was computed as in various previous works^15,38,39^ using a Gaussian weighting kernel with a user-defined standard deviation *w*. Additionally, the truncated version of the LCC gradient was used^15^ in order to speed up the computation. This approximation has been reported^15^ to outperform the full gradient approach. Although the results of this work are obtained using the LCC metric, it should be pointed out that our method is generally applicable to other similarity metrics as well. The automatic time-step and penalty weight estimation allows for a seamless transition between different similarity metrics without the need to adjust other algorithm hyperparameters.

The proposed optimization method is summarized in Algorithm 1. In order to avoid local minima and speed-up the computation, a coarse-to-fine scheme^40^ was used where the registration is performed on a number of pyramid levels determined by the requirement that no dimension becomes smaller than 16 pixels. Before downsampling, a Gaussian smoothing filter is applied on the images with a standard deviation of $(s-1)/2$ where *s* denotes the scaling factor (so that larger SD is used for lower resolutions to avoid aliasing). At the final resolution level ($s=1$) (original image resolution) no smoothing filter is applied. The values of the registration parameters $f^i$ at each level are used to initialize the parameters for the subsequent level. We used linear interpolation for resampling the images and the registration parameters $f^i$.

The size of the time step *t* is crucial to ensure convergence and satisfaction of the solenoidal constraint. Since numerical instabilities are usually a result of large values of the update terms $t \delta {\mathscr {D}} / \delta f^i$ in Eq. 7, we selected an automatically estimated time step that ensures boundedness of those terms given by

10 $$\begin{aligned} t = 0.02/ \max _{\vec {x} \in \Omega } \bigg \{ \sqrt{ \bigg (\frac{\delta {\mathscr {D}}}{\delta f^1} \bigg ) ^2 + \bigg ( \frac{\delta {\mathscr {D}}}{\delta f^2} \bigg ) ^2 + \bigg ( \frac{\delta {\mathscr {D}}}{\delta f^3} \bigg ) ^2 + \bigg (\frac{\delta {\mathscr {D}}}{\delta f^4} \bigg ) ^2 } \bigg \}. \end{aligned}$$

The prefactor of 0.02 was empirically found to deliver good numerical stability and convergence speed. The penalty weight $\theta$ associated to the solenoidal constraint in Eq. 6 is also empirically related to the time step *t* by $\theta = 0.4/t$. In this way, both *t* and $\theta$ do not require any external input.

Due to the use of the chain rule in Eq. 9, numerical errors in the similarity metric gradient $\delta {\mathscr {D}} / \delta u^i$ tend to be amplified and propagate to the estimation of the $\delta {\mathscr {D}} / \delta f^i$ update terms. This also affects the time step estimation in Eq. 10. In order to ameliorate this effect and reduce numerical instabilities, we used outlier pruning, mapping outliers of $\delta {\mathscr {D}}/\delta f^i$ to their nearest non-outlier value. Our simple outlier detector defines outliers as elements more than three standard deviations from the mean. We found that this computationally efficient method strikes a good balance between accuracy and speed. Despite the additional step of outlier pruning, this methods enables the use of larger time steps therefore accelerating convergence in addition to making the optimization more stable.

**Algorithm 1:**
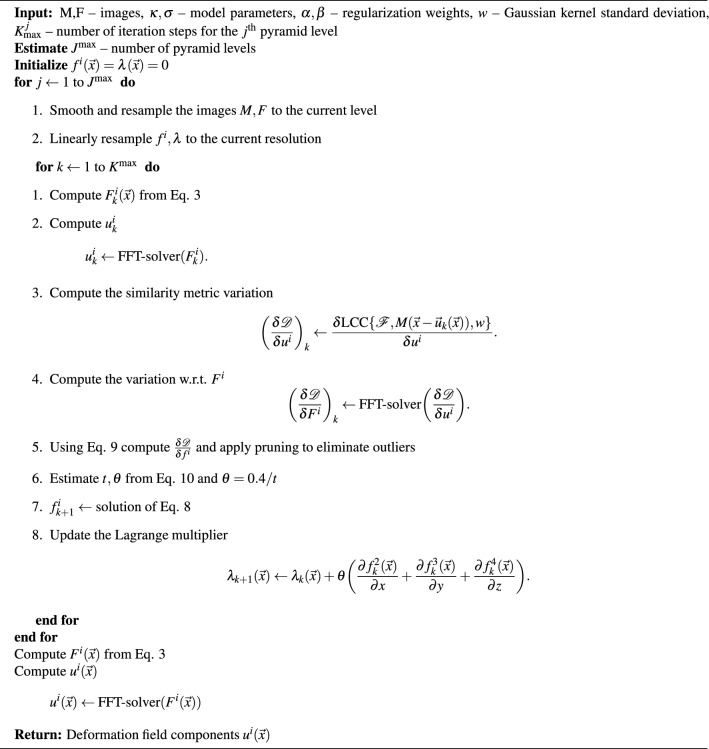
ALM for generalized div-curl algorithm

## Experimental results

In this section we describe a series of experiments that are designed to test the performance of the proposed GDC algorithm on a set of problems with different underlying physical assumptions.

### Effect of the curl regularization on the constraint convergence

Both the solenoidal constraint (Eq. 4) and the curl regularizer affect the estimation of the curl components $f^i$ $(i=2,3,4)$. In this experiment we aim to investigate their interplay by examining the constraint convergence profile for different regularization parameters $\kappa$ and $\beta$. A single image pair was used from the DIR-lab dataset^41^ (case 4) in which the initial deformation was moderate, corresponding to an average displacement of 9.42 mm. We made this choice because for this experiment we want to carry out the registration on a single resolution level without the employment of a coarse-to-fine scheme, in order to eliminate the influence of image and control parameter resampling. The images were thus registered at a fixed resolution of $64\times 64\times 32$. To evaluate the degree of satisfaction of our equality constraint we used the mean residue $\langle \vert {\mathcal {C}}_k (\vec {x}) \vert \rangle _{x \in \Omega }$ where ${\mathcal {C}}_k$ is the expression of the constraint 4 evaluated at iteration *k*. The domain $\Omega$ was taken to be the pulmonary volumes defined by manual segmentations. This was done in order to avoid measurements in the image background which are devoid of physical meaning and because we expect that the effect of parameter variation on the convergence profile will be more pronounced in this region where larger scale motion takes place. We also conducted the same experiment after disabling the constrained optimization component by setting $\theta = 0$. In this way, we were able to get some insight to the effect of the ALM framework on the optimization.

**Figure 1:**
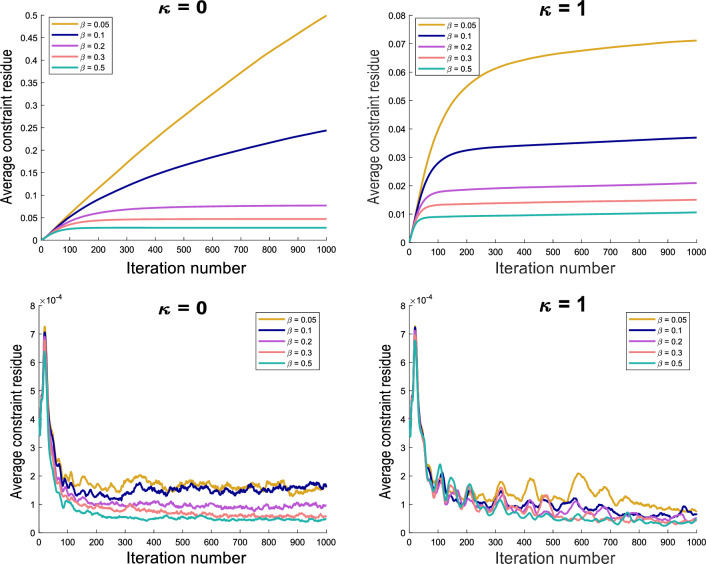
Summary of the constraint profile for different curl regularization parameters with and without the introduction of the ALM framework. The y-axis shows the average constraint residue inside the pulmonary volumes. Top left: First order curl regularization ($\kappa = 0$) without constrained optimization ($\theta =0$). Top right: Second order curl regularization ($\kappa = 1$) without constrained optimization ($\theta =0$). Bottom left: First order curl regularization ($\kappa = 0$) with constrained optimization ($\theta \ne 0$). Bottom right: Second order curl regularization ($\kappa =1$) with constrained optimization ($\theta \ne 0$).

The results of the experiment are shown in Figure 1. We make the following observations:

1. Without the introduction of constrained optimization the quantity $\langle \vert {\mathcal {C}}_k (\vec {x}) \vert \rangle$ increases monotonically with the iteration number. Therefore, as expected, without the explicit introduction of a constrained optimization method like the ALM, the constraint is not satisfied and the $f^i$ (i=2,3,4) do not admit the interpretation of the curl components since they have a non-trivial divergence.
2. For $\kappa = 1$ the corresponding values of $\langle \vert {\mathcal {C}}_k (\vec {x}) \vert \rangle$ are smaller than for $\kappa = 0$. This is because $\kappa =1$ imposes a penalty on the derivatives of $f^i$ (i=2,3,4) which appear in the expression of ${\mathcal {C}}_k (\vec {x})$. Furthermore, for the same $\kappa$, increasing $\beta$ values lead to smaller average residue due to the stricter penalty imposed on $f^i$ (i=2,3,4).
3. With the introduction of constrained optimization (bottom plots), the average residue is decreased by about two to three orders of magnitude compared to the unconstrained optimization (top plots).
4. For constrained optimization, larger $\beta$ values lead to smaller residue for the same reason as the unconstrained case.
5. Using constrained optimization (bottom plots) the final average residue does not significantly vary for different $\kappa$ and $\beta$ values as is the case for unconstrained optimization (top plots).
6. For $\kappa = 1$ the average residue takes more iterations before it stabilizes. This effect is more pronounced for smaller $\beta$ values.

### Pulmonary registration

Lung registration has been an active field of research, presenting some additional challenge due to the high degree of compressibility of lung tissue. Many registration algorithms align visible structures very well but have been reported to do so at the expense of anatomical plausibility. Most notably, the deformation fields are often not bijective, resulting in negative values of the Jacobian determinant $\text {Jac}(\vec {u}) := \det ({\mathbb {I}}+\nabla \vec {u} )$. Similarly, significant spatial irregularities of $\text {Jac}(\vec {u})$ are considered implausible. Some methods^42,43^ have successfully attempted to avoid such artefacts by explicitly adding an infinite penalty for transformations with $\text {Jac}(\vec {u}) \rightarrow 0$ or $\text {Jac}(\vec {u}) \rightarrow \infty$, leading to deformations of high accuracy and plausibility. We here wanted to test whether our framework allows for sufficient spatial accuracy while maintaining anatomical properties such as the smoothness of $\text {Jac}(\vec {u})$. To that end, we configured our proposed regularization with $\kappa = \sigma =1$ (corresponding to the second order div-curl regularizer). We used the 10 publicly available DIR-LAB datasets^41^. All images were resampled to a resolution of $256 \times 256 \times 128$. We registered the two extreme respiratory phases of each dataset for varying values of $\alpha$. The regularization weight associated to the curl smoothness was kept to a small value of $\mu = 0.1$. To assess the algorithm accuracy, we measured the average target registration error (TRE) defined as

$$\begin{aligned} \text {TRE} = \left \langle \sqrt{(u-u_0)^2 + (v-v_0)^2 + (w-w_0)^2} \right \rangle , \end{aligned}$$

where $(u_0,v_0,w_0)$ are the Cartesian components of the ground truth deformations and (*u*, *v*, *w*) the estimated ones. Angle brackets are used to denote the average with respect to the points for which the ground truth deformation is known. For further assessment, we also compute the standard deviation of the logarithm of Jacobian determinant (SDLogJac) inside the lungs to evaluate the smoothness of the volumetric changes.

**Figure 2:**
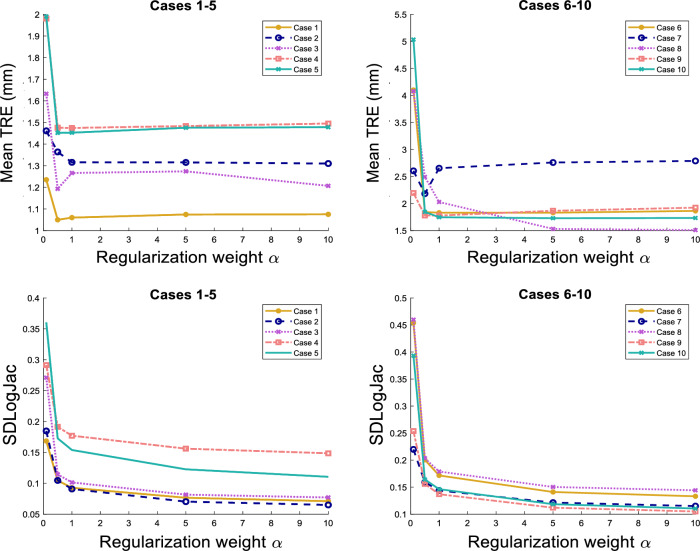
Summary of the pulmonary registration experiment results. The average TRE and SDLogJac is reported for different values of the regularization weight $\alpha$. Cases 1-5 and 6-10 are shown separately for clearer illustration.

**Figure 3:**
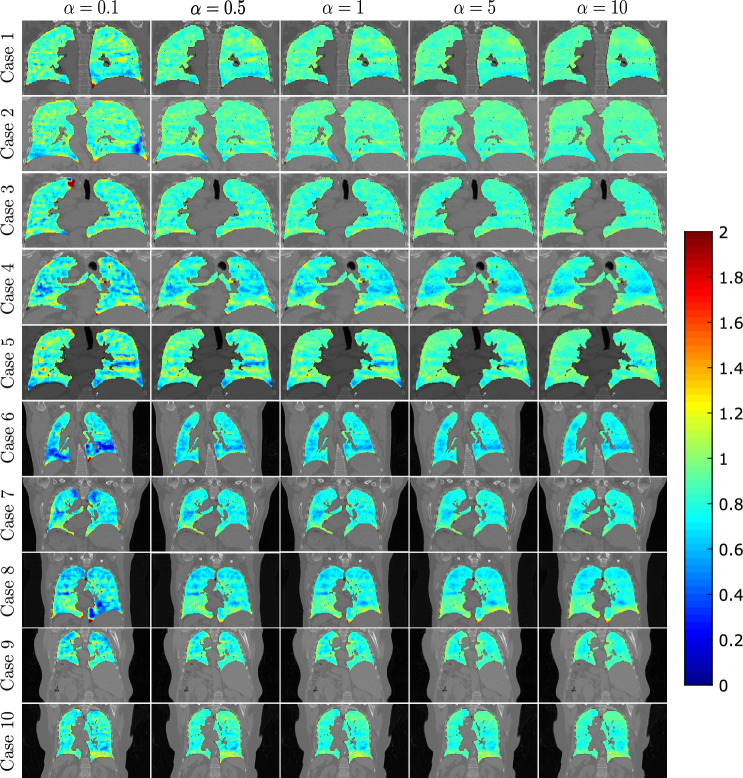
Coronal slices of all 10 cases for different $\alpha$ values showing the reference anatomy with the Jacobian determinant overlaid on it.

The results are shown in Figure 2. The TRE and SDLogJac are reported separately for cases 1-5 and 6-10. We observe that although the TRE remains stable for a large range of regularization weight values, the SDLogJac decreases. Additionally, the optimal TRE was below 1.5 mm for cases 1-5 and below 2 mm for all cases except case 7 for which the lowest TRE was 2.18 mm. This indicates that the proposed algorithm can consistently achieve subvoxel accuracy. To visually illustrate the effect of varying the regularization weight $\alpha$ on the Jacobian determinant, we have collected coronal slices from all the cases in Figure 3 showing the spatial distribution of the Jacobian determinant for the different $\alpha$ values.

### Locally incompressible motion inside rigid organs

A number of previous studies have examined the performance of registration algorithms inside the liver and kidneys using constraints on the Jacobian determinant^44,45^ or registration using a pre-defined region of interest (ROI)^46^. On a similar approach, a method for organ motion estimation from raw image data has been proposed^47^ using diffeomorphic incompressible flows by projecting the velocity fields on the space of divergence-free vector fields. It is expected that inside the liver and kidneys the Jacobian determinant of the image transformation is approximately equal to 1. Therefore, significant deviations from this value are interpreted as physically implausible. In this experiment we used four pairs of abdominal T1w MR-scans acquired at different time points, with misalignment caused by respiratory and peristaltic motion. The resolution of the images is $192 \times 75 \times 192$ with a voxel size of $1.95 \times 2 \times 1.95$ mm$^3$. The liver and kidneys on each scan have been segmented by a medical expert. We demonstrate here that our model can be easily tuned for this task by imposing a variable penalty on the divergence. Therefore for this task we set $\sigma =0$ (corresponding to a first order div regularizer) and $\kappa = 1$. The regularization weight $\beta$ associated to the curl was kept to a moderate value of $\beta = 2$ and we varied the divergence related weight $\alpha$ over a range of different values (0.1,0.5,1,2,5,10) in order to obtain different degrees of (local) rigidity in the estimated transformation fields. In order to assess the registration quality we evaluated the Dice similarity coefficient (DSC) after registration on the liver and kidneys. As a measure of the local incompressibility inside the liver and kidneys we used $\vert \text {Jac} (\vec {u})-1 \vert$. In this measurement, we excluded points located on the organ boundaries in order to avoid numerical errors due to boundary effects such as sliding motion.

**Figure 4:**
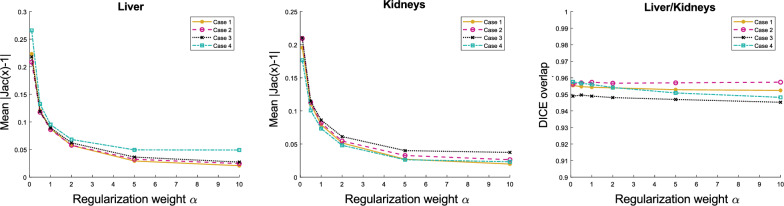
Left: Mean $\vert \text {Jac}(\vec {u})-1\vert$ inside the liver ploted against the regularization weight $\alpha$ associated to the divergence regularization for the 4 different cases tested. Middle: Same plot for the kidneys. Right: DSC scores after registration computed on the liver and kidney volumes for each case and for each regularization weight value.

**Figure 5:**
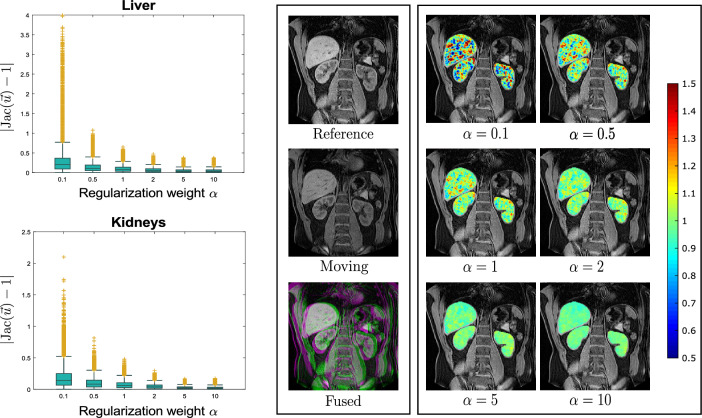
Detailed analysis and visual illustration of the Jacobian determinant distribution inside the liver and kidneys for the abdominal case with the largest initial displacement (case 4). The boxplots illustrate the distribution of $\vert \text {Jac}(\textbf{u}) -1 \vert$ inside the liver (upper boxplot) and kidneys (bottom boxplot) for different values of the regularization weight $\alpha$. In the middle, coronal slices of the reference and moving image are shown together with their superposition. On the right, coronal slices of the reference image are shown with the Jacobian determinant colormaps overlaid for different values of the regularization weight $\alpha$.

The mean values of $\vert \text {Jac}(\vec {u})-1 \vert$ for each case are shown in Figure 4 and a detailed analysis for one of the cases is shown in Figure 5. The DSC after registration indicate that all registrations were successful in aligning the organ volumes. Additionally, the plots in Figure 4 indicate that an increase in the regularization weight $\alpha$ results in a higher degree of incompressibility. The same conclusion can be drawn from Figure 5 where the distributions of $\vert \text {Jac}(\vec {u})-1 \vert$ inside the liver and kidneys are shown to cluster closer to zero as $\alpha$ increases. These results are in accordance with our theoretical analysis.

### Synthetically deformed pelvic image

In this experiment, our goal was to test the efficacy of the curl regularization of the proposed framework. For this reason, we set up an experiment entailing the estimation of irrotational motion. We artificially deformed a pelvic MR scan of resolution $256 \times 256 \times 128$ and voxel size $1.7 \times 1.7 \times 0.7$ mm$^3$. The synthetic deformation was generated by displacing 1000 randomly selected control points located inside the bladder. The entire deformation field was interpolated using B-splines. The control points were radially displaced from a pre-defined geometric center which was taken to be the center of the bladder. In this way, the generated deformations are almost irrotational providing a fitting testing setup for the proposed curl regularization. We coregistered the original and deformed images using a fixed second order div-regularization ($\sigma =1, \alpha = 1$) and a curl regularization ($\kappa = 0$) for a number of different weights $\beta$. We measured the TRE and the curl magnitude inside the bladder for the different regularization weight values.

**Figure 6:**
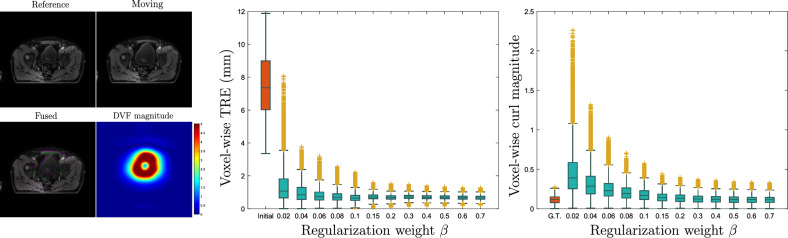
Left: The original (reference) and deformed (moving) MR images together with the deformation magnitude. Middle: Boxplots of the voxel-wise TRE for various values of the regularization parameter $\beta$. The initial TRE boxplot is shown in orange. Right: Boxplots of the curl magnitude for different $\beta$ values. The ground truth (G.T.) curl magnitude boxplot is shown in orange. Both the TRE and the curl magnitude are measured exclusively inside the bladder.

The results are summarized in Figure 6. As we can see, increasing the regularization weight $\beta$, decreases the magnitude of the curl (left boxplot). At the end, for $\beta = 0.7$ the curl magnitude boxplot becomes almost identical to the ground truth (G.T.) and the corresponding TRE attains its minimum value. This demonstrates that highly irrotational motion can be recovered using a strong penalty on the curl magnitude. More generally, adjusting the regularization weight $\beta$, we can easily model our prior knowledge regarding the degree of irrotationality of the motion field.

### Thoracic registration with large initial displacement

In order to further assess the accuracy of the proposed GDC framework and enable future comparisons with other methods, we used the 30 cases provided by the Lung CT DIR dataset^48^. Each case contains the inhale/exhale thoracic CT scans together with a large number of vessel-bifurcation landmarks (on average 1260 landmarks per case). The scans have been collected from a variety of repositories, were acquired using different acquisition parameters, include both healthy subjects and subjects with lung disease and have varying voxel sizes. Those factors contribute to significant data heterogeneity. One of the advantages of this study is that the generated landmarks are uniformly distributed throughout the lung volume, offering a more holistic evaluation of alignment accuracy.

For each case, the inhale and exhale scans were resampled to an isotropic voxel size of 1mm. The images were then padded to ensure that their dimensions agree and further padding was added to avoid crucial structures such as the lungs being close to image boundaries. Subsequently, both images were resampled to $256 \times 256 \times 256$ voxels. For consistency, the algorithm parameters used in this experiment were the same as the ones used in the pulmonary registration experiment of the DIR-lab cases. We used a second order div-curl model ($\sigma = \kappa =1$) with $\mu = 0.1$ and $\alpha = 5$ (which is the midpoint of the range of values used in the pulmonary registration experiment in Figure 2).

**Figure 7:**
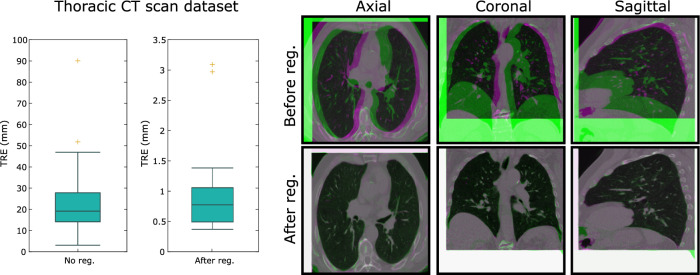
Left: Boxplots of the target registration error before and after registration for the 30 thoracic CT scans. The largest TRE values before registration are often due to rigid misalignment. Right: An example (case 16) of the image overlap before and after registration. The moving image is shown in green and the reference image in magenta. For this case, the TRE is reduced from 34.8 mm before registration to 1.38 mm after registration.

The results are shown in Figure 7. On the same figure, we have included a visualization of one of the cases (case 16) to illustrate the degree of alignment before and after registration. This case represents one of the largest residual TRE of 1.38 mm (corresponding to the whisker of the “after registration” boxplot). The mean (standard deviation) of the TRE’s is 0.91 mm (0.65 mm). The two worst cases were registered with a TRE close to 3 mm while in all the rest, the proposed method achieved a TRE scores below 1.5 mm.

## Discussion

In this work we have proposed a highly-flexible physics-inspired solution for deformable image registration. At its core, the problem formulation relies on the Helmholtz decomposition of a vector field into its divergence and curl components, which allowed placing independent penalties on their values and/or their derivatives. This resulted in a framework encompassing a wide spectrum of physical models that can be configured by tuning the differential operator orders $\kappa ,\sigma$ in Eq. (1). We have tested our model on a number of anatomies with different physical properties such as incompressibility or irrotationality and demonstrated that our framework can deliver deformations which are both of high accuracy as well as conform with physical model constraints. Beyond these specific cases, the proposed solution provides a generalized framework that can be easily adjusted to different tissue types and be combined with a multitude of image similarity metrics. The high adaptability of our framework lends itself to a convenient general purpose registration tool that could be advantageous in clinical practice where the use of multiple distinct and specialized registration solutions is often not feasible.

Aside from the flexibility of the regularization model, a significant advantage of our method is that while in the scope of this work we have used the LCC as a data fidelity term, it is worth noting that the employed numerical solver described in Algorithm 1 allows for its seamless replacement with other similarity metrics such as sum of squared differences or normalized gradient fields. This, in turn, facilitates the extension of the proposed registration model to a wider category of, potentially multi-sensor, problems. Furthermore, this also allows for a straight-forward exploration of various data fidelity - regularization combinations, via the same numerical framework, and subsequent selection of an optimal model for the application-at-hand.

An important benefit of decomposing the regularization in terms of the divergence and curl components is the reduced sensitivity to parameter selection. Using regularizations such as smoothness, that penalize all degrees of freedom indiscriminately, the output deformation field can be highly sensitive to regularization weight variation. On the contrary, disentangling the motion parameters related to volume change and rotation, allows for varying the penalty on one set of parameters without affecting the other, leading to more robust and reproducible motion estimation. This can be seen from the TRE plots in Figure 2 where a change of the divergence related regularization weight by an order of magnitude has practically no effect on the spatial accuracy of the motion fields in spite of the Jacobian determinant distribution being substantially affected (Figures 2 and 3). The same conclusion can be drawn from the abdominal experiment, where the DSC overlap of the liver and kidneys remains stable for different regularization weights (Figure 4) but the distribution of $\text {Jac}(\vec {u})$ inside the organs drastically changes. Those findings also highlight the fact that when using contrast based evaluation metrics such as landmarks or organ segmentations, the physical properties of the obtained deformations are often overlooked since the registration accuracy is only assessed on a small contrast-rich subset of the entire anatomy of interest. Previous studies have emphasized this point and have proposed the use of biologically motivated quality assurance metrics beyond anatomical landmarks to enable more holistic evaluations^49^. Such information is essential for tasks requiring voxel-wise accuracy in contrast deficient regions.

With respect to the convergence properties of the solenoidal constraint, as can be seen in Figure 1, the average residue reaches a plateau after the first 100-150 iteration steps, indicating that the constraint does not further decrease from this point on. We have empirically found this to be a sufficient amount of iterations per resolution level for both the constraint residue and the registration to converge. In effect, the computation time for our current Matlab implementation is approximately 15 minutes for a $256 \times 256 \times 128$ image. The computational bottleneck rests in the FFT-solver which is used twice in every iteration of the algorithm (steps 2 and 4 in the k-iteration loop of Algorithm 1). The first time, it is used to map the div-curl components back to the Cartesian coordinate frame (which is necessary to warp the moving image and compute the similarity metric gradient) and the second time it is used to compute the similarity metric gradient with respect to $F^i$. Those two steps account for approximately 40% of the total computation time. Due to the highly localized computational burden and the inherent parallelizability of the GDC algorithm, we believe that the computation time could be largely reduced. We are confident that such improvements could render the GDC algorithm more suitable for applications with restrictive latency requirements.

It should also be stressed out that the proposed optimization framework can be applied to a much larger family of regularizers. Due to the linearity of Eq. (8), our framework can be used to solve the much more general registration problem given by the following functional:

11 $$\begin{aligned} \min _{ u^i } \; \bigg \{ \int {\mathscr {D}} + \sum _{m=1} ^M \alpha _m \left\| \nabla ^{\sigma _m} \text {div} \; \vec {u} \right\| ^2 + \sum _{n=1} ^N \beta _n \left\| \nabla ^{\kappa _n} \text {curl} \; \vec {u} \right\| ^2 \; d \vec {x} \bigg \}, \end{aligned}$$

where an arbitrary number of divergence or curl orders are penalized with different weights. In this work we elected to only focus on the use of two terms on the basis of clarity of presentation. Due to the generality of regularizers that the framework of Eq. (11) incorporates, we believe that it can be useful for addressing complex models in the future. Another point that deserves some attention is that although integer values of $\sigma _m$ and $\kappa _n$ in Eq. (11) admit a clear interpretation, their physical meaning becomes less transparent for fractional values. As discussed in previous studies^9,36^ differential operators of fractional order are not well-defined in the spatial domain but can be viewed as implementing features of both contiguous integer values. This conclusion is corroborated by our experience. We further point out that the use of the div-curl variables as registration parameters could serve as a useful tool for addressing more general regularization functionals of the form ${\mathcal {R}}(\text {div}(\textbf{u}),\text {curl} ( \textbf{u}))$ for which the resulting equations of motion are possibly non-linear in the divergence and curl components. One such example would be a penalty on the L1-norm of the div-curl components which would allow for spatial discontinuities. An approach along these lines was proposed^50^ for the 2D case and reported the potential of preserving large shearing values on a small subset of the field of view comprising organ boundaries. Allowing for such motion types would be an interesting extension of our work that we intend to investigate further in the future.

In spite of enabling greater modeling flexibility, employing a large number of regularization terms in Eq. (11) introduces further weights whose tuning can require additional effort. To this end, we believe that future work is needed in the direction of automatic parameter calibration by extracting physical quantities from image data. This could facilitate not only a faster algorithm calibration but also allow for more accurate subject-specific registration models. Recent work in this direction using deep learning methods^51^ has shown promising results, maintaining good accuracy while improving the physical plausibility of the estimated deformation fields. Finally, the spatial invariance of the regularization weights across images encompassing anatomic structures of very different physical properties is a strong assumption that bears no physical justification. It would therefore be advantageous to allow for spatially varying regularization weights that can account for the different mechanical properties of the various anatomical regions. Previous studies have addressed this issue to some extent by imposing local anatomical constraints^52^ or by adapting the regularization based on the position of a voxel relative to organ boundaries using segmentations^17,53,54^ or image-based information^55^. Although these approaches offer some degree of local control, we believe that further investigation is needed in order to model the full complexity of anatomical structures and their interactions.

Closing, we would like to underline that the application domain of our method is not restricted to medical image registration. For example, our physics inspired framework is highly applicable in image sequence analysis of fluid flows. The extraction of physically consistent fluid flows can be be of major scientific interest in environmental sciences such as oceanography and meteorology where monitoring the atmosphere is becoming increasingly significant. What is more, we believe that an important message of the present work is that the adoption of proper optimization variables that are fittingly adapted to the problem at hand, can be crucial to the solvability of an optimization problem. We believe that this rationale could be conducive to efficiently solving a number of other optimization problems.

## Conclusions

We have presented a novel optimization solution for physically-inspired image registration regularization, based on a generalized div-curl model. We have demonstrated that the generality of our approach allows convenient adaptation to different registration problems across anatomies/organs with a variety of underlying physical properties. Given the increasing need for high accuracy computational methods in the medical field, we believe that the introduction of physically informed motion models can be highly advantageous in future applications. Finally, we hope that the proposed optimization solution using the divergence and curl components as registration parameters, can provide a fitting setup for other optimization problems.

## Data Availability

The two thoracic CT image datasets used in this study for the validation of pulmonary registration and convergence analysis are available at www.dir-lab.com and zenodo.org/records/8200423. The rest of the image datasets can be shared upon reasonable request to the corresponding author.
